# Obesity-associated family with sequence similarity 13, member A (FAM13A) is dispensable for adipose development and insulin sensitivity

**DOI:** 10.1038/s41366-018-0222-y

**Published:** 2018-10-09

**Authors:** Jiazhen Tang, Hongyi Zhou, Khushboo Sahay, Wenqiong Xu, Jing Yang, Wei Zhang, Weiqin Chen

**Affiliations:** 1 Department of Endocrinology and Metabolism, The First Affiliated Hospital of Nanchang University, Nanchang, Jiangxi Province 330006 China; 2 Department of Physiology, Medical College of Georgia at Augusta University, Augusta, GA 30912 USA; 3 Department of Comparative Biosciences, University of Illinois at Urbana-Champaign, Urbana, IL 61802 USA; 4 Department of Respiratory Medicine, The First Affiliated Hospital of Nanchang University, Nanchang, Jiangxi Province 330006 China

**Keywords:** Fat metabolism, Fatty acids

## Abstract

**Background:**

Obesity and its associated morbidities represent the major and most rapidly expanding world-wide health epidemic. Recent genome-wide association studies (GWAS) reveal that single nucleotide polymorphism (SNP) variant in the Family with Sequence Similarity 13, Member A (*FAM13A*) gene is strongly associated with waist–hip ratio (WHR) with adjustment for body mass index (BMI) (WHRadjBMI). However, the function of FAM13A in adipose development and obesity remains largely uncharacterized.

**Methods:**

The expression of FAM13A in adipose tissue depots were investigated using lean, genetic obese and high fat diet-induced obese (DIO) animal models and during adipocyte differentiation. Stromal vascular cells (SVCs) or 3T3-L1 cells with gain and loss of function of FAM13A were used to determine the involvement of FAM13A in regulating adipocyte differentiation. Adipose development and metabolic homeostasis in *Fam13a*^−*/*−^ mice were characterized under normal chow and high fat diet feeding.

**Results:**

Murine FAM13A expression was nutritionally regulated and dramatically reduced in epididymal and subcutaneous fat in genetic and diet-induced obesity. Its expression was enriched in mature adipocytes and significantly upregulated during murine and human adipogenesis potentially through a peroxisome proliferator-activated receptor-gamma (PPARγ)-dependent mechanism. However, *Fam13a*^*−/−*^ mice only exhibited a tendency of higher adiposity and were not protected from DIO and insulin resistance. While *Fam13a*^*−/−*^ SVCs maintained normal adipogenesis, overexpression of FAM13A in 3T3-L1 preadipocytes downregulated β-catenin signaling and rendered preadipocytes more susceptible to apoptosis. Moreover, FAM13A overexpression largely blocked adipogenesis induced by a standard hormone cocktail, but adipogenesis can be partially rescued by the addition of PPARγ agonist pioglitazone at an early stage of differentiation.

**Conclusions:**

Our results suggest that FAM13A is dispensable for adipose development and insulin sensitivity. Yet the expression of FAM13A needs to be tightly controlled in adipose precursor cells for their proper survival and downstream adipogenesis. These data provide novel insights into the link between FAM13A and obesity.

## Introduction

Obesity and its associated health comorbidities is a worldwide epidemic which imposes a serious economic and health burden on society. Genetic factors have been attributed to around 40–70% of inter-individual variability in body mass index (BMI) which is commonly used to assess overall obesity. Overall adiposity and body fat distribution are both heritable traits and well-established predictors of adverse metabolic outcomes [1, 2]. Recent genome-wide association studies (GWAS) have successfully identified a large number of obesity-associated genetic loci. Among these, 97 gene variants are associated with overall adiposity measured by body mass index (BMI) [3] and 49 gene variants are associated with fat distribution measured by waist circumference (WC) and waist to hip ratio (WHR) [4]. However, the majority of these variants have not been characterized. Their biological functions and pathological relevance to adipose biology and obesity remain unknown.

A recent GWAS study with the largest cohort identified a genetic variant (rs9991328) in the Family with Sequence Similarity 13, Member A (*FAM13A*) gene to be highly associated with WHR adjusted BMI (WHRadjBMI) [4], suggesting FAM13A may play a role in body fat distribution.FAM13A expression was abundant in human adipose tissue [5]. The expression of *FAM13A* gene was shown to be associated with adipose morphology, specifically adipocyte cell numbers (hyperplasia) [6]. Single nucleotide polymorphisms (SNPs) in *FAM13A* have been widely associated with chronic obstructive pulmonary disease (COPD) [7–9], asthma severity [10], lung cancer [11] as well as pulmonary fibrosis [12], underscoring an important involvement of FAM13A in human lung disease etiology. The COPD risk allele at the *FAM13A* locus was shown to be associated with increased expression of FAM13A in human lung samples [13]. The biological function of FAM13A in lung physiology remains elusive. FAM13A was found to form complex with protein phosphatase 2A and β-catenin, and exhibited different effects on the stability of β-catenin in different experimental settings, suggesting that FAM13A is likely a context-dependent regulator of the Wnt pathway [13, 14]. Most recently, FAM13A was identified to promote fatty acid oxidation (FAO) possibly by interacting with and activating Sirtuin 1 (Sirt1), thereby increasing the expression of CPT1α as well as other β-oxidation genes in lung [15]. Despite its association with fat distribution, to date, no study has explored the potential role of FAM13A in regulating adipogenesis and obesity. In this study, we investigated the expression and function of FAM13A in adipose development and metabolic homeostasis in vitro and in vivo.

## Materials and methods

### Animal experiments

All animal procedures were approved by Augusta University’s Institutional Animal Care and Use Committee (IACUC). Mice were maintained under standard conditions with controlled 12 h/12 h-light–dark cycle and 21 ± 1 °C room temperature. *Lep*^*ob/ob*^, *Lepr*^*db/db*^ and C57BL/6 J mice were obtained from Jackson Laboratory (Bar Harbor, ME). *Fam13a*^*−/−*^ mice were backcrossed to C57BL/6 (Stock#: 027, Charles River) for seven generations and genotyped as previously reported [14]. 4–5 week old *Fam13a*^*+/+*^ and *Fam13a*^*−/−*^ littermates were fed with high fat diet (HFD, D12492, 60% Kcal from fat, Research Diets, NJ) for up to 12 weeks. Mice were not randomized. Six week old C57BL/6 J male mice were simply randomized into two experimental groups for ad libitum access to low fat control diet (LFD, D12450B, 10% Kcal from fat, Research Diets, NJ) and 60% HFD for up to 12 weeks as reported [16]. All mice studies were not blinded. All mice were sacrificed after 4 h fasting with isoflurane.

### Human tissues

Human adipose tissues were collected from obese (BMI > 40) patients undergoing gastric bypass surgeries. The study protocol was approved by the Institutional Review Boards of the Medical College of Georgia at Augusta University and informed consent was obtained from the subjects.

### Cell culture, plasmids, transient transfection, luciferase assay

All cell culture reagents were obtained from Fisher Scientific Inc. (Pittsburgh, PA). Insulin, dexamethasone, isobutylmethylxanthine (IBMX), puromycin were obtained from Sigma-Aldrich (St. Louis, MO). The 3T3-L1 (ATCC CL-173) and NIH/3T3 cells (ATCC CRL-1658) were cultured following ATCC instructions. pLightSwitch-Luc vector harboring predicted murine *Fam13a* promoter was obtained from Active Motif (Carlsbad, CA). pSV Sport PPARγ2 was obtained from Addgene (#8862). Promoter-driven luciferase activities were analyzed in NIH/3T3 cells by using LightSwitch™ Luciferase Assay System with or without PPARγ2 overexpression.

### Retrovirus and lentivirus production and infection

pBABE-puro-PPAR gamma2 vector was obtained from Addgene (#8859). Retroviral packaging Bosc-23 cells were cotransfected with the targeting plasmid and packaging vector pCL-eco (Imgenex, Sorrento Valley, CA). Lentiviral particles were produced by transient transfection of 293 T cells with a packaging plasmid psPAX2 and envelope plasmid pMD2.G together with pSin-EF2-Puro vector alone or pSin-EF2-FAM13A-FLAG-Puro. 48 h after transfection, the culture media containing the virus particles were collected and mixed with DMEM 10% FBS at 1:1 to infect 3T3-L1 preadipocytes or NIH-3T3 fibroblasts in the presence of 8 μg/ml polybrene. Cells were then selected with 2 µg/ml puromycin for at least 4 days before analysis or differentiation.

### MTT assay, Edu proliferation and immunofluorescence microscopy

Cell number was determined by Vybrant MTT cell proliferation assay kit (Invitrogen, Grand Island, NY) following manufacturer’s instruction. Immunofluorescence staining against FAM13A was performed as previously described [17]. For proliferation, same amount of cells were plated and grown on coverslips. Cells were labeled with Edu for 24 h by Click-iT™ EdU Alexa Fluor™ 488 Imaging Kit (ThermoFisher Scientific) as instructed. Cells were then counter-stained with Dapi and analyzed using fluorescent microscope. Cells from six random fields were counted for each treatment and the proliferation rate was defined as (no. of fluorescein-labeled nuclei)/(no. of Dapi nuclei) × 100.

### FACS analysis of apoptosis

Cells with and without serum starvation for 24 h were fixed and permeabilized. Cells were then stained with APO-BrdU TUNEL Assay Kit with Alexa Fluor 488 anti-Brdu (A23210, ThermoFisher Scientific) and LIVE/DEAD Fixable for Far Red Dead Cell Stain Kit, for 633 nm excitation (L34973, ThermoFisher Scientific). Apoptosis was assessed by FACS analysis using FACSCalibur (Becton Dickinson) under 488 nm and 635 nm lasers. Cells negative for both far-red dye and TUNEL staining are live cells; far-red-dye negative, TUNEL-positive staining cells are early apoptotic cells; far-red-dye positive, TUNEL-negative staining cells are dead cells; far-red-dye positive, TUNEL-positive staining cells are primarily cells in late stages of apoptosis. The total percentage of early and late apoptotic cells were compared.

### Adipose tissue fractionation, adipocyte differentiation, triacylglycerol (TAG) content measurement and oil-red O (ORO) staining

Mouse or human adipose tissues were digested with collagenase (Sigma Aldrich), filtered, and centrifuged to separate mature floating adipocytes from the pelleted stromal vascular fraction (SVF) as described previously [18]. Preadipocytes rich SVF pellets and 3T3-L1 cells were cultured to confluence and induced to differentiate into mature adipocytes by addition of insulin, dexamethasone, and IBMX (DMI) as previously described [18]. At indicated days, cells were washed and directly dissolved in PBS containing 1% triton X-100. The intracellular TAG in the adipocytes was measured with an Infinity Triglyceride assay kit (ThermoFisher Scientific). The TAG content was normalized to the amount of cellular protein as determined using a Bradford  protein assay (Bio-Rad) and expressed as per milligram of protein. ORO staining was performed and photographed with camera or under microscopy as previously described [18].

### Plasma biochemistry

Plasma nonesterified fatty acid (NEFA) (Wako Chemicals USA Inc.), glycerol (Sigma-Aldrich), total cholesterol, total triacylglycerol levels (Fisher Scientific Inc.) were measured colorimetrically.

### Histology, body composition, glucose tolerance and insulin tolerance tests

Tissue samples were fixed, processed, and stained with hematoxylin and eosin (H&E) and visualized under microscopy. Fat and lean body masses were measured by NMR-based Bruker minispec LF90II (Bruker company, German). Insulin tolerance tests were performed in mice fasted for 6 h and then injected intraperitoneally (i.p.) with human insulin (Humulin; Novo Nordisk) at 0.75 U/kg for mice on normal chow diet, and 2.5 U/kg for mice on HFD. Glucose tolerance tests were performed in 6 h fasted chow-fed mice injected by i.p. or 16 h fasted HFD-fed mice administered by gastric gavage with glucose (1.5 g/kg body weight). Blood glucose levels were measured by One-touch Ultra glucose meter before and at 15, 30, 60, and 120 min after administration.

### RNA isolation and real-time quantitative PCR

Total RNA was isolated from tissues or cells with TRIzol (Invitrogen) and reverse transcribed using MLV-V reverse transcriptase with random primers (Invitrogen). Real-time quantitative RT-PCR was performed on a Stratagene MX3005. Data were normalized to two housekeeping genes (*Ppia* and *36B4*) based on Genorm algorithm and expressed as fold changes relative to control cells or tissues.

### Immunoblot analysis

Tissues and cells were homogenized and lysed in lysis buffer as previously described [19]. The protein concentration was determined by Bradford  protein assay (Bio-Rad). Equal amounts of proteins were loaded and immunoblot analysis was carried out according to standard protocol. The blots were visualized using the ECL chemiluminescence system by Amersham Imager 600 (GE healthcare) and quantified by densitometric analysis using ImageQuantTL (GE healthcare). The following antibodies were used: rabbit polyclonal antibodies against FAM13A (HPA038109; Sigma Life Science); PPARγ (2435), β-Catenin (8480), IRS1 (2382), GSK3β (12456), pGSK3β^S9^ (9336), SIRT1 (9475) from Cell Signaling Technology; GAPDH (60004-1-IG, Proteintech); PLIN1 (GP29; Progen Biotechnik GmbH); ATGL (10006409; Cayman Chemical); C/EBPα (sc-61, Santa Cruz); β-ACTIN (MAB1501) from Millipore.

### Statistical analysis

All in vitro experiments were performed in triplicate for at least three times. Animal experiments were performed in at least two independent cohorts. Detailed sample sizes for animal studies were provided in each figure legend. Sample sizes were chosen by power analysis based on pilot studies. Statistical analyses of the data were analyzed by SigmaPlot 13 (Systat Software, Inc) using a two-tailed unpaired *t* test with equal variance. Data were presented as the mean ± SEM with statistical significance set at a *P* value of <0.05.

## Results

### FAM13A is nutritionally regulated and downregulated in genetic and diet-induced obesity

**Fig. 1:**
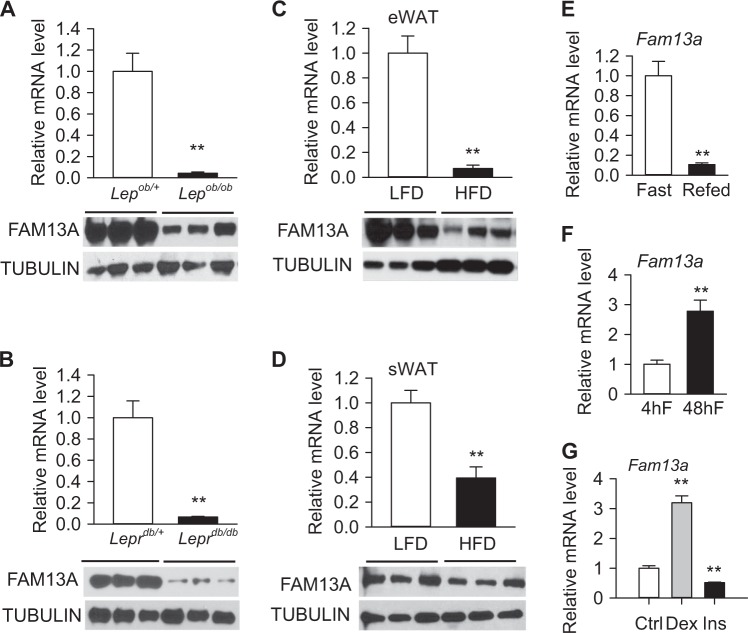
FAM13A is nutritionally regulated and downregulated in genetic and diet-induced obesity. **a**, **b** Gene (*n* = 6/group) and protein expression (*n* = 3/group) of FAM13A in epididymal white adipose tissue (eWAT) of 3 months old male genetic obese (**a**) *Lep*^*ob/ob*^ and (**b**) *Lepr*^*db/db*^ mice as compared to their lean countparts. **c**, **d** Gene (*n* = 10/group) and protein (*n* = 3/group) expression of Fam13a in (**c**) eWAT and (**d**) inguinal subcutaneous WAT (sWAT) of low fat diet (LFD) and high fat diet (HFD)-fed male C57BL/6 J mice. **e**, **f** mRNA expression of *Fam13a* in eWAT of 10 weeks old male C57BL/6 J mice after (**e**) a 18 h fast and 6 h refeed; (**f**) 4 h fast (4hF) as compared to 48 h prolonged fasting (48hF) (*n* = 7/group). **g** mRNA expression of *Fam13a* in mature 3T3-L1 adipocytes after 18 h treatment with dexamethasone (Dex) and insulin (Ins) as compared to vehicle-treated control (Ctrl) cells. Representative data were shown from 3 independent experiments in triplicate. **p < 0.005

The significant association of SNPs near the *FAM13A* gene locus with WHRadjBMI suggests FAM13A may play a role in regulating adipose tissue distribution. We first examined the expression of FAM13A in adipose tissue of murine obese models. Surprisingly, we found FAM13A expression in epididymal white adipose tissue (eWAT) of genetic obese *Lep*^*ob/ob*^ and *Lepr*^*db/db*^ mice were dramatically downregulated at both mRNA and protein levels as compared to their lean counterparts (Fig. 1a, b). Likewise, the mRNA and protein expression of FAM13A in eWAT and inguinal subcutaneous WAT (sWAT) were markedly reduced in our previously reported HFD-induced obese mouse model [16] as compared to LFD-fed lean mice (Fig. 1c, d). These data underscore a dramatic decrease of FAM13A in obese adipose tissue.

Given the significant downregulation of FAM13A in adipose tissue of animal models with hyperphagia, we then asked whether FAM13A is nutritionally regulated. We found that *Fam13a* mRNA was markedly reduced in eWAT of 6 h refed C57BL/6 J mice as compared to overnight (18 h) fasted mice (Fig. 1e). Conversely, *Fam13a* transcript was upregulated (~3-fold) in eWAT of C57BL/6 J mice after prolonged 48 h food deprivation as compared to mice after a 4 h fast (Fig. 1f). To further confirm *Fam13a* gene is a nutritionally responsive gene in adipose tissue, we treated 3T3-L1 mature adipocytes with dexamethasone and insulin to mimic fasting and refeeding responses. We found *Fam13a* was upregulated by catabolic hormone dexamethasone but inhibited by insulin treatment (Fig. 1g), similar to a well-known catabolic gene adipose triglyceride lipase (ATGL) [20]. Collectively, these data demonstrate that *Fam13a* expression in adipose tissue is dynamically regulated in response to changes in the nutritional status.

### FAM13A is upregulated during adipocyte differentiation potentially through PPARγ

**Fig. 2:**
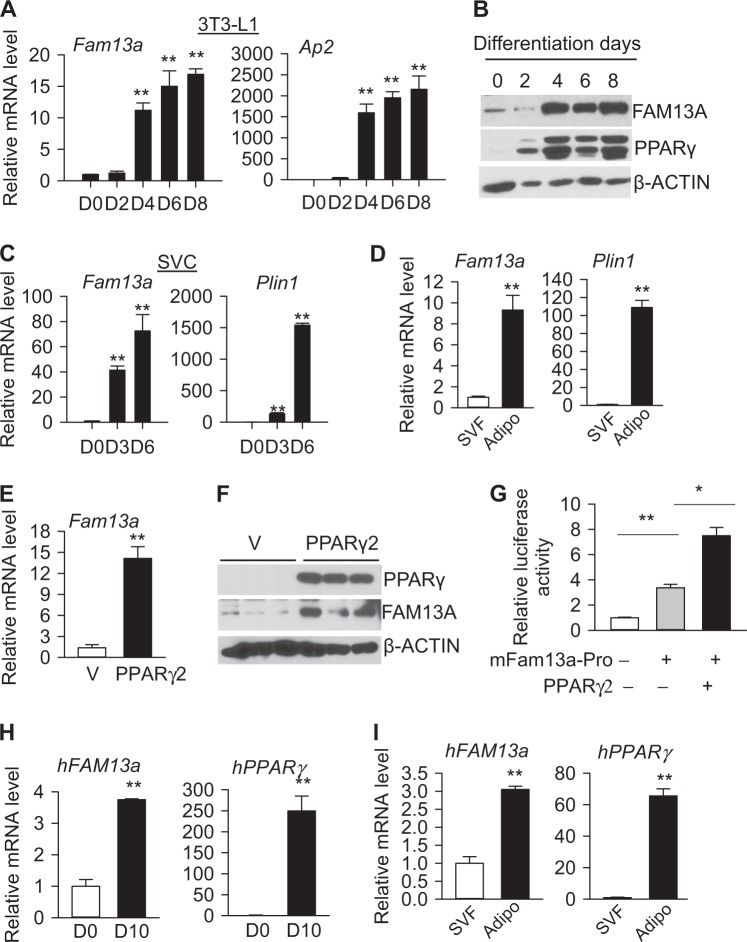
*Fam13a* is upregulated during adipocyte differentiation potentially through PPARγ. **a**, **b** Time course of *Fam13a* (**a**) mRNA and (**b**) protein expression during induced differentiation of 3T3-L1 cells by DMI. The expression of *Ap2* mRNA and PPARγ protein was used as positive controls. **c** Time course of *Fam13a* and *Plin1* mRNA expression during induced differentiation of mouse primary stromal vascular cells (SVCs) by DMI. **p < 0.005 vs D0. **d** mRNA expression of *Fam13a* and *Plin1* in stromal vascular fraction (SVF) and adipocytes (Adipo) fractionated from eWAT of 10 week old male C57BL/6 J mice (*n* = 6). **e**, **f** mRNA (**e**) and protein expression (**f**) of FAM13A in NIH/3T3 cells stably transduced with empty lentiviruses (V) or lentivirus overexpressing PPARγ2. **g** The pLight-switch luciferase reporter construct harboring predicted murine *Fam13a* promoter were transfected into NIH/3T3 cells together with PPARγ2. 48 h after transfection, cells were harvested for luciferase assays. Reporter activity is expressed relative to the transfection with empty plasmid (set to 1). Representative data from 3 independent experiments were shown. **h**, **i** mRNA expression of *FAM13A* and *PPARγ2* (**h**) during induced differentiation of human primary stromal vascular cells and (**i**) in SVF and adipocytes fractions from sWAT of human adipose tissue. *p < 0.05; **p < 0.005

To further analyze the role of FAM13A in adipose biology, we first studied the expression of FAM13A during adipocyte differentiation of 3T3-L1, a well-established adipocyte differentiation model. At both mRNA and protein levels, FAM13A was gradually upregulated during the course of preadipocyte differentiation into mature adipocytes and reached the highest level in mature adipocytes (Fig. 2a, b). The expression of *Ap2* and PPARγ were used as markers for adipogenesis. Similarly, we found that *Fam13a* gene expression was upregulated during adipocyte differentiation of murine primary stromal vascular cells (SVCs) (Fig. 2c). Its expression was also highly enriched in fractionated adipocytes as compared to stromal vascular fractions (SVF) (Fig. 2d). These data clearly suggest that FAM13A exhibits an adipocyte-specific expression signature.

To identify the potential mechanisms underlying FAM13A upregulation during adipogenesis, we next generated stable NIH-3T3 cells overexpressing PPARγ2, the master transcription factor governing adipocyte differentiation. *Fam13a* mRNA expression was elevated ~14-fold in NIH-3T3 cells stably transduced with pBABE-puroPPARγ2 retrovirus as compared to vector infected cells (Fig. 2e). Western blot confirmed the overexpression of PPARγ2 and the induction of FAM13A protein (Fig. 2f). Furthermore, we performed a luciferase assay in NIH/3T3 cells using pLight-Switch murine *Fam13a*-pro, a luciferase reporter driven by *Fam13a* promoter. Overexpression of PPARγ2 significantly upregulated *Fam13a* promoter activity (Fig. 2g). These data suggest that PPARγ2 is upstream of FAM13A expression. Moreover, *FAM13A* gene expression was also markedly upregulated during human preadipocyte differentiation into mature adipocytes (Fig. 2h) and enriched in mature adipocyte fraction as compared to SVF isolated from human subcutaneous adipose tissue (Fig. 2i). Taken together, we conclude that FAM13A is regulated by the master adipogenic transcription factor PPARγ, and its expression is gradually increased during murine and human adipocyte differentiation.

### Loss of FAM13A minimally affects adiposity, diet-induced obesity and metabolic homeostasis

**Fig. 3:**
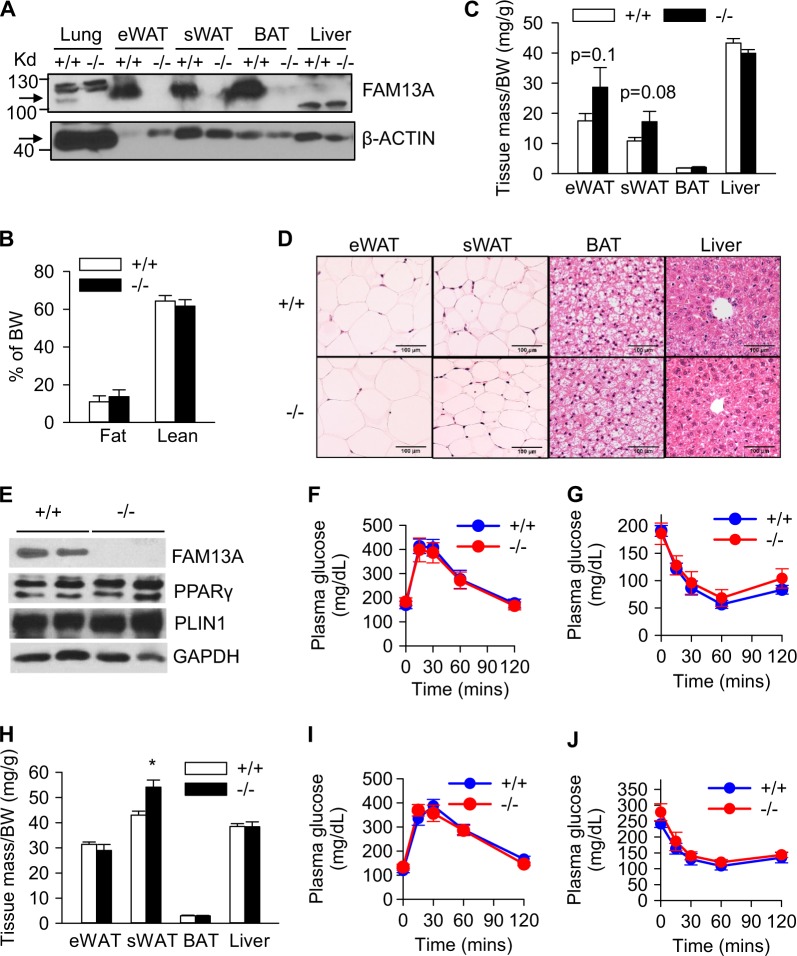
Loss of *Fam13a* minimally affects adiposity, diet-induced obesity and metabolic homeostasis in vivo. **a** FAM13A protein expression in various murine tissues. **b** Fat and lean masses presented as % of body weight. **c** Tissue masses (mg) as normalized to BW (**g**). *n* = 6–7/group. **d** Representative H&E images of eWAT, sWAT, BAT and liver. Scale bar, 100 μm. **e** Representative western blot of FAM13A and adipose markers PPARγ and PLIN1 in eWAT, *n* = 4/group. **f**, **g** Glucose tolerance test (**f**) and (**g**) insulin tolerance test. Chow fed 14–15 week old male *Fam13a*^*+/+*^ (+/+) and *Fam13a*^*−/−*^ (−/−) mice were used for experiments performed in **a**. **g** *n* = 6–7/group. **h** Tissue masses (mg) as normalized to BW (**g**) after 20 weeks of HFD feeding. *n* = 5–7/group. **i**, **j** Glucose tolerance test (**i**) and insulin tolerance test (**j**) on mice fed with HFD for 11 weeks and 13 weeks respectively (*n* = 6–7 per group)

To dissect roles of FAM13A in regulating adipose tissue development and distribution, we first analyzed the protein expression of FAM13A in lung, liver, and various adipose depots. We observed that the expression levels of FAM13A in eWAT, sWAT and brown adipose tissue (BAT) were much higher as compared to that in lung; while no FAM13A was detected in liver (Fig. 3a). As expected, FAM13A was not detectable in tissues from *Fam13a*^*−/−*^ mice (Fig. 3a). Body weight, circulating levels of plasma TAG, cholesterol, NEFA and glycerol were comparable between the wild type and *Fam13a*^*−/−*^ male and female mice (Table S1). Fat mass and lean mass were also similar between 16 week old *Fam13a*^*+/+*^ and *Fam13a*^*−/−*^ mice (Fig. 3b). After dissection, *Fam13a*^*−/−*^ mice did demonstrate a tendency towards higher masses of eWAT and sWAT as compared to *Fam13a*^*+/+*^ mice (Fig. 3c), but maintained normal histological appearance of eWAT, sWAT, BAT and liver (Fig. 3d). Western blot confirmed similar expression of adipose tissue marker proteins (PPARγ and PLIN1), further suggesting lack of overt difference in WAT of *Fam13a*^*−/−*^ mice (Fig. 3e). Consistent with this, *Fam13a*^*−/−*^ SVCs can differentiate into mature adipocytes to a similar degree as its wild-type counterparts, evidenced by comparable mRNA expression of PPARγ and Ap2 at D0 and D8 adipocytes (Fig. S1a), similar protein expression of PPARγ and PLIN1 in D8 mature adipocytes (Fig. S1b), as well as ORO stained neutral lipids (Fig. S1c). Besides, *Fam13a*^*−/−*^ mice also displayed no obvious perturbations in whole body glucose tolerance and insulin sensitivity (Fig. 3f, g, respectively). Notably, lack of overt changes in *Fam13a*^*−/−*^ adipocytes and mice was not due to compensatory upregulation of its known paralog genes (*Fam13b* and *Fam13c*) (Fig. S1b and Fig. S2f respectively). These data indicate despite the abundant expression of FAM13A in mature adipocytes, loss of FAM13A exerts little impact on adipocyte differentiation, adiposity and metabolic homeostasis.

When subjected to high fat diet, *Fam13a*^*−/−*^ mice retained similar body weight gain (Fig. S2a) and fat composition (Fig. S2b). There was a slight (19%) increase in sWAT mass in *Fam13a*^*−/−*^ mice as compared to *Fam13a*^*+/+*^ mice (Fig. 3h). However, no significant difference in adipocyte sizes was detected between mutant and control animals (Fig. S2c), and *Fam13a*^*−/−*^ mice also maintained similar glucose tolerance (Fig. 3i) and insulin resistance (Fig. 3j) as compared to their wild-type counterparts under HFD. These data further suggest a minimal effect of FAM13A deficiency in adipose tissue distribution and whole-body insulin action.

### Overexpression of FAM13A suppresses preadipocyte survival through repressing β-catenin signaling

**Fig. 4:**
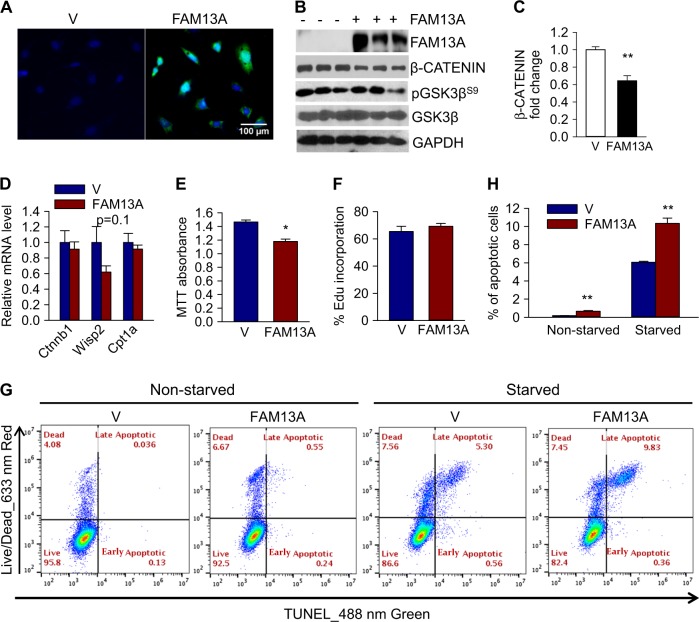
Overexpression of murine FAM13A impairs preadipocyte survival. **a** FAM13A immunofluorescent staining, scale bar, 100 μm. **b**, **c** western blot (**b**) and quantification (**c**) of β-catenin expression. **d** qPCR analysis. **e** MTT viability assay. **f** Edu incorporation in vector (V) and FAM13A-overexpressing lentivirus transduced 3T3-L1 preadipocytes. **g** The levels of apoptosis evaluated by FACS analysis following APO-BrdU TUNEL staining combined with LIVE/DEAD cell staining in vector (V) and FAM13A-overexpressing lentivirus transduced 3T3-L1 preadipocytes with or without 24 h serum starvation. The picture shows one of the three experiments. **h** Numerical results of early and late apoptotic cells from FACS analysis. Values are presented as mean ± SEM in 3 separate experiments *p < 0.05; **p < 0.005 vs V

To further dissect the function of FAM13A in adipogenesis, we next generated 3T3-L1 preadipocytes stably overexpressing a full-length 117 kD murine FAM13A and confirmed its overexpression by both immunofluorescence (Fig. 4a) and western blot (Fig. 4b). Interestingly, the expression of β-catenin protein (Figs 4b, c), but not its encoded gene *Ctnnb1* (Fig. 4d) was reduced in FAM13A-overexpressing (OE) preadipocytes as compared to control (vector)-transduced cells. There was a tendency of lower mRNA expression of Wnt1-inducible-signaling pathway protein 2 (*Wisp2*), frequently used as a marker of canonical Wnt activation [21] in FAM13A-OE preadipocytes (Fig. 4d), supporting reduced β-catenin signaling. The canonical Wnt pathway is essential for the survival of adipose precursors by regulating IGF1 expression [22]. Indeed, following plating of equivalent numbers of lentivirally-transduced 3T3-L1 preadipocytes, FAM13A-OE preadipocytes displayed lower cell numbers based on MTT assay (Fig. 4e). There was no difference in cell proliferation between vector and FAM13A-OE preadipocytes (Fig. 4f). However, FAM13A-OE 3T3-L1 preadipocytes were more susceptible to apoptosis which was further exacerbated after serum starvation (Fig. 4g, h). These data underscore a pro-apoptotic act of FAM13A potentially through downregulating β-catenin signaling in adipose precursor cells.

### Excessive FAM13A expression inhibits early-stage adipocyte differentiation which could be rescued by PPARγ agonist

**Fig. 5:**
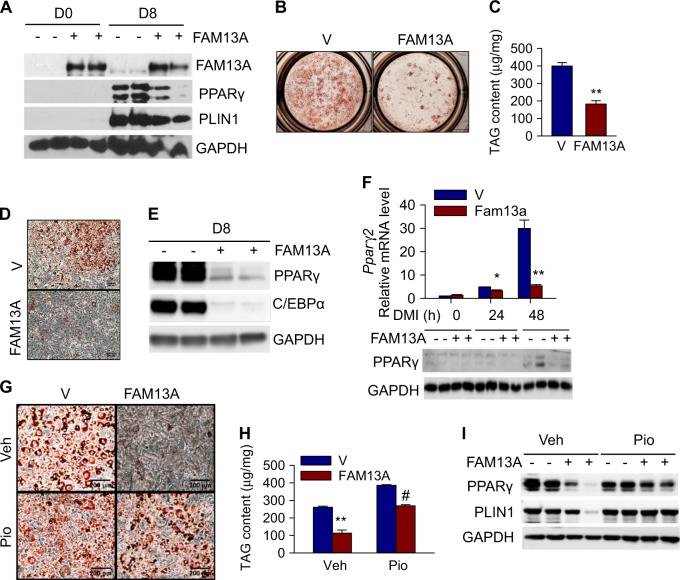
Excessive expression of FAM13A inhibits adipocyte differentiation. **a** Same number of cells were initially plated and cultured to confluence for DMI induced differentiation. Western blot at 2 days after confluence (D0) and 8 days (D8) after DMI induction. **b**, **c** ORO staining (**b**) and (**c**) intracellular TAG content in D8 vector and FAM13A-OE adipocytes. **d**, **e** 25% more FAM13A-OE cells were plated to normalize cell numbers at confluence for differentiation. **d**, **e** ORO staining (**d**) and protein expression (**e**) at D8 after DMI induction. **f** *Pparγ* mRNA and protein expression at 0, 24 h and 48 h after DMI stimulation. **g** ORO staining. **h**, **i** TAG content (**h**) and protein expression (**i**) at D8 after DMI induction with addition of vehicle (Veh) or 1 µM pioglitazone (Pio) from D0–D4. *p < 0.05; **p < 0.005 vs V; #*p* < 0.05 vs Veh

We further examined whether ectopic expression of FAM13A in adipose precursor cells impact downstream adipogenesis. When cells were cultured to confluence and subjected to adipocyte differentiation, FAM13A overexpression markedly inhibited adipocyte differentiation as evidenced by impaired protein expression of PPARγ and PLIN1 (Fig. 5a), reduced ORO staining (Fig. 5b) and diminished intracellular TAG content (Fig. 5c) in day 8 (D8) mature adipocytes. mRNA expression of adipocyte marker genes including *Pparγ*, *C/ebpα* and *Plin1*, were comparable at D0 (2 days after confluence) but significantly repressed at D8, whereas the preadipocyte marker gene *Pref1* was upregulated about 4-fold in D8 FAM13A-OE mature adipocytes (Fig. S3a–d, respectively). Notably, the level of lentiviral-expressed FAM13A at D0 was about 10 times higher than the endogenous level of FAM13A in D8 mature adipocytes (Fig. 5a). These data suggest an anti-adipogenic effect of FAM13A when overexpressed at super-physiological level. Growth arrest at confluence appears to be a prerequisite for subsequent differentiation [23]. To analyze whether the anti-adipogenic effect of FAM13A is due to reduced survival rate in preadipocytes which may cause uneven confluent status before subjecting to adipocyte differentiation, we plated 25% more FAM13A-OE preadipocytes to ensure both groups reach confluence simultaneously. However, FAM13A-OE preadipocytes were again unable to differentiate into adipocytes based on reduced ORO staining (Fig. 5d) and blunted expression of adipocyte marker proteins (Fig. 5e). Mitotic clonal expansion (MCE) during the first 2 days of differentiation is a prerequisite for terminal adipocyte differentiation [24]. Considering the effect of FAM13A overexpression on preadipocyte survival, we also analyzed MCE and found similar expansion in cell numbers between Vector and FAM13A-OE cells within 48 h of adipogenesis (Fig. S3e), suggesting MCE was not the direct cause of impaired differentiation in FAM13A-OE cells. Further examination of FAM13A-OE cells identified blocked upregulation of the master adipogenic transcription factor PPARγ as early as 24 h after DMI stimulation (Fig. 5f). Addition of pioglitazone together with DMI for the first 4 days of differentiation could partially rescue adipogenic deficiency in FAM13A-OE cells by restoring TAG contents (Fig. 5g, h) and adipocyte marker protein expression (Fig. 5i). Thus, FAM13A overexpression in adipose precursor cells not only impairs survival but also interferes with the normal transcriptional cascade of adipogenesis during fat cell differentiation.

## Discussion

Previous study has identified *Fam13a* as one of the top ten genes that are dramatically downregulated in adipose tissue of diet-induced obese animals [25]. In this study, we further confirmed this finding in both genetic and diet-induced obese animal models. We also identified *Fam13a* as an adipose tissue abundant gene highly induced by fasting (dexamethasone) and suppressed by refeeding (insulin), which may partially explain the remarkable downregulation of *Fam13a* in hyperphagic obese animals. Downregulation of *Fam13a* in obese adipose tissue could also be attributed to increased adipose inflammation as IL-1β has been previously shown to suppress *Fam13a* expression [26].

Our study not only identified a striking upregulation of FAM13A during human and murine adipogenesis, but also provided the first evidence for FAM13A as a downstream target of PPARγ during adipocyte differentiation (Fig. 2). Indeed, murine *Fam13a* promoter contains a potential PPRE binding site (data not shown). Despite its upregulation by PPARγ during adipogenesis, loss of FAM13A exerts no effect on fat cell differentiation (Fig. S1). It also displayed minimal effect on fat distribution under both chow and high fat diet (Fig. 3). As the master regulator of adipogenesis, PPARγ regulates the expression of numerous genes which collectively control adipogenesis. Knockout of PPARγ target genes individually may not always cause detectable phenotypes. While ablation of FAM13A alone is insufficient for causing an adipose development phenotype, we cannot completely rule out the possibility that forced expression of FAM13A in preadipocytes may play a role during adipogenesis (Fig. 5). Clearly, future studies are still needed to fully understand roles of FAM13A in adipose development.

While our manuscript was in the final stage of preparation, Wardhana DA et al. reported similar regulation of *Fam13a* during adipogenesis. They also found no changes in adiposity in their *Fam13a*^*−/−*^ mice under both chow and high fat diets [27]. However, their *Fam13a*^*−/−*^ mice were insulin resistant and glucose intolerant due to adipose IRS1 downregulation and ATGL upregulation [27]. In our study, we found the expression of IRS1 and ATGL was similar between differentiated wild-type and *Fam13a*^*−/−*^ adipocytes (Fig. S1c) or eWAT and sWAT of normal chow diet (NCD)-fed wild type and *Fam13a*^*−/−*^ mice (Fig. S2d–g). Consistently, *Fam13a*^*−/−*^ adipocytes or NCD-fed *Fam13a*^*−/−*^ mice showed neither differences in response to insulin (Fig. S1d and Fig. 3g, respectively), nor changes in basal and β3-adrenergic receptor agonist (CL316,243)-stimulated lipolysis as compared to their respective control groups (data not shown). There was a tendency toward lower IRS1 expression in eWAT and sWAT of *Fam13a*^*−/−*^ mice after HFD feeding (Fig. S2d–g), which did not result in worsened insulin sensitivity and glucose tolerance (Fig. 3i–j). It is not clear whether these discrepancies could be attributed to the differences in diet fat contents or the genetic backgrounds of *Fam13a*^*−/−*^ mice between two studies. Meanwhile, we found no changes in SIRT1 protein and *Cpt1α* mRNA expression in either *Fam13a*-deficient or overexpressed adipose precursor or differentiated adipocytes (Figs. S1e, S3f and S4d, respectively), suggesting FAM13A is dispensable for fatty acid oxidation in adipose depots. Further studies will be required to rigorously test whether FAM13A deficiency affects other adipocyte function in response to metabolic stresses yet to be characterized.

The WHRadjBMI-associated rs9991328 SNP in FAM13A is different from all other SNPs linked to human chronic lung diseases [28]. While COPD-linked SNPs cause higher FAM13A protein expression in lung [13], whether the rs9991328 SNP is linked to abnormal FAM13A expression in human adipose tissue is completely unknown. Importantly, a positive association of FAM13A expression with lung tumor cell proliferation and survival was recently reported [29]; and FAM13A gene expression was also found to exert a stimulatory effect on human adipose hyperplasia [6]. While these findings clearly indicate an important role of FAM13A in regulating tumor or (pre)adipose cell numbers, they seem to be opposite to our finding which suggests a negative role of FAM13A in regulating the survival of adipose precursors. The relationship between FAM13A expression and adipose hyperplasia in humans awaits further study. Notably, FAM13A overexpression in 3T3-L1 preadipocytes reduced total β-catenin protein expression, confirming its role in promoting β-catenin degradation [13]. The canonical Wnt pathway has been shown to play an important anti-apoptotic role and is essential for the survival of adipose precursors [22]. Hence, it is conceivable to observe an increased apoptosis in our FAM13A-OE 3T3-L1 preadipocytes (Fig. 4g, h). FAM13A has been shown to affect recruitment of PP2A which modulates GSK3β phosphorylation thus β-catenin proteasome degradation [13]. Yet, we failed to identify difference in GSK3β phosphorylation in FAM13A-OE preadipocytes (Fig. 4b), suggesting the presence of other regulatory pathways. Nevertheless, our data underscore the important role of FAM13A in regulating adipose precursor cell numbers potentially through mitigating β-catenin signaling.

Wnt/β-catenin pathway represents a major axis upon which various signals converge to influence PPARγ activation and preadipocyte differentiation [30]. Several studies suggest inhibition of Wnt/β-catenin signaling in preadipocytes stimulates differentiation [31–33]. Surprisingly, despite reduced β-catenin signaling, FAM13A overexpression did not promote adipogenesis. Instead, it blocked early-stage adipogenesis through blunting PPARγ upregulation, suggesting the presence of a β-catenin-independent pathway that plays a more dominant role in regulating adipogenesis. Notably, the overexpressed 117 kD isoform of FAM13A contains a Ras homologous GTPase-activating protein (RhoGAP) domain which potentially mediates Rho GTPase signaling [26, 29], an important pathway that regulates PPARγ and adipogenesis [34]. Future study is needed to dissect whether FAM13A mediates RhoGTPase signaling thus adipogenesis.

The physiologic and/or clinical relevance of FAM13A overexpression in regulating adipose precursor cell survival and adipogenesis is not clear at this point. It is worth to mention that adipose-specific *Fam13a*-Tg mice lack changes in adipose tissue mass [27], inconsistent with the anti-adipogenic role of FAM13A. This discrepancy could result from the fact that FAM13A overexpression is relatively limited to mature adipocytes not adipose precursors in aP2-promoter driven *Fam13a*-Tg mice. Thus, animal model in which FAM13A is overexpressed specifically in adipose precursor cells is desirable. Comparison of FAM13A expression and the survival of preadipocytes from adipose tissue of lean and obese humans (carrying rs9991328 SNP at FAM13A) would be ideal. Nevertheless, our data implicate the importance of maintaining temporal expression of *Fam13a* gene at correct stages of adipose development.

In conclusion, our data pinpoint a dispensable role of FAM13A in adipogenesis and argue against FAM13A as a positive regulator of adipose insulin sensitivity. But forced expression of FAM13A in adipose precursor cells modulates cell survival and early adipogenesis. Hence, our results provide insights to further elucidate mechanisms through which FAM13A contributes to adipose biology.

## Electronic supplementary material

Supplemental material
