# Supplementary material for: Obesity-associated family with sequence similarity 13, member A (FAM13A) is dispensable for adipose development and insulin sensitivity
Source: Int J Obes (Lond). 2018 Oct 9;43(6):1269–80. doi: 10.1038/s41366-018-0222-y (PMC6456441; doi:10.1038/s41366-018-0222-y)
Supplement: Supplementary file 1 — Supplemental material [file 41366_2018_222_MOESM1_ESM.docx]

**Supplemental Data**

**Figure S1. Deletion of *Fam13a* does not affect adipocyte differentiation and β-catenin signaling *in vitro*. a** RT-PCR of *Fam13a* and adipocyte marker genes and **b** RT-PCR of *Fam13a* paralogs (*Fam13b* and *Fam13c*) in *Fam13a^+/+^* (+/+) and *Fam13a^−/−^* (-/-) SVCs at 2 days after confluence (D0) and 8 days (D8) after DMI stimulation. **: p< 0.005 vs +/+; #: p< 0.05 vs D0. **c-d** Protein expression (**c**) and ORO staining (**d**) of D8 *Fam13a^+/+^* and *Fam13a^−/−^* adipocytes. **e** AKT phosphorylation at Serine 473 in response to 100 nM insulin stimulation in D8 *Fam13a^+/+^* and *Fam13a^−/−^* adipocytes. **f** Protein expression at D0 and D8 *Fam13a^+/+^* and *Fam13a^−/−^* adipocytes.

**Figure S2. FAM13A deficiency does not prevent diet-induced obesity and impair adipose tissue insulin signaling. a** Growth curves during the course of 12 weeks high fat diet (HFD) feeding starting from 4-5 weeks old. **b** % of fat and **c** H&E staining of eWAT and sWAT of *Fam13a^+/+^* (+/+) and *Fam13a^−/−^* (-/-) mice fed with HFD for 20 weeks respectively. n=5-7/group. **d-g** Western blot and quantification of IRS1 and ATGL expression as normalized to GAPDH in eWAT **(d-e)** and sWAT **(f-g)** of *Fam13a^+/+^* and *Fam13a^−/−^* mice under normal chow diet (NCD) and HFD (n=5). **f** RT-PCR analysis of *Fam13a* paralogs (*Fam13b* and *Fam13c*) in eWAT of *Fam13a^+/+^* and *Fam13a^−/−^* mice under both NCD and HFD (n=5).

**Figure S3. FAM13A overexpression blocks adipogenesis. a-d** RT-PCR analysis of *Pparγ2* (**a**), *C/ebpα* (**b**), *Plin1* (**c**) and *Pref1* (**d**) in vector and FAM13A lentivirus-transduced 3T3-L1 preadipocytes at 2 days after confluence (D0) and 8 days (D8) after DMI induction. *: p< 0.05; **: p< 0.005 vs D0; #: p< 0.05 vs V. **e** Cell numbers at 0, 24 and 48 h after DMI induction. **f** Protein expression in D0 and D8 vector (V) and FAM13A-OE cells at D0 and D8.

**Supplemental Table 1. Phenotypic comparison of wild-type and *Fam13a* deficient mice**

|  | Male | | Female | |
| --- | --- | --- | --- | --- |
| **Genotype** | ***Fam13a^+/+^*** | ***Fam13a^−/−^*** | ***Fam13a^+/+^*** | ***Fam13a^−/−^*** |
| BW (g) | 29.5 ± 0.9 | 31.6 ± 0.8 | 21.6 ± 0.8 | 23.3 ± 0.6 |
| TAG (mg/dL) | 36.5 ±2.3 | 33.5 ± 0.9 | 26.2 ± 1.2 | 30.9 ± 2.7 |
| TC (mg/dL) | 112 ± 8 | 105 ± 10 | 77.0 ± 3.6 | 74.0 ± 1.6 |
| NEFA (mM) | 0.86 ± 0.1 | 0.8 ± 0.05 | 0.70 ± 0.05 | 0.75 ± 0.12 |
| Glycerol (mg/dL) | 29.8 ± 2.3 | 34.1 ± 2.7 | 29.3 ± 2.0 | 26.8 ± 3.8 |
| Mice (14-16 weeks of age) were fed with normal chow diet. Plasma were collected after 4 h fast. Data were presented as means ± SEM. TAG, triglycerides; TC, total cholesterol; NEFA, nonesterified free fatty acid. N=5-6/group. | | | | |
